# Evaluation of the Relationship of Sleep Disturbances to Severity and Common Behaviors in Autism Spectrum Disorder

**DOI:** 10.21203/rs.3.rs-2674526/v1

**Published:** 2023-03-23

**Authors:** Stacy Miner, Molly McVoy, Elizabeth Damato

**Affiliations:** Kent State University; Case Western Reserve University School of Medicine, University Hospitals Cleveland Medical Center; Case Western Reserve University

**Keywords:** Autism spectrum disorder, sleep disturbances, social and communication skills, behavior, clinical assessment

## Abstract

**Background:**

Autism spectrum disorder (ASD) is one of the most puzzling disorders of childhood. Recent research of comorbidities that accompany ASD and are commonly attributed to the diagnosis, indicate that they may contribute to the severity of behavioral symptoms of the disorder.

Disturbed sleep in all children can decrease cognition, decrease focus, increase performance problems, and alter mood and behavior. Children with ASD experience an increased sensitivity to disturbed sleep that may increase the severity of the disorder. Disturbed sleep patterns, such as increased sleep latency, nighttime waking and early arousal, have been identified in up to 80% of children with ASD. This study explored the relationship of disturbed sleep and the severity of the core ASD symptoms.

**Methods:**

Actigraphy and an accompanying sleep diary captured disturbed sleep patterns in 24 children, ages 6–12, with ASD. Participants wore a GT3X actigraphy monitor for 7 nights to collect data on patterns of disturbed sleep. Parents completed a sleep diary and the Autism Spectrum Rating Scale (ASRS) questionnaire.

A descriptive analysis was used to report the characteristics of nighttime sleep and sleep efficiency as well as sleep disturbances. Pearson’s r determined the relationships between the number of sleep disturbances and the severity of ASD behavioral scores and diagnostic severity (determined by the ASRS).

**Results:**

Of the 24 study participants, almost 92% had one or more sleep disturbances. A positive correlation was present between the number of sleep disturbances and the severity of delays in social and communication symptoms. A moderate effect size was found between the number of sleep disturbances and unusual behaviors in ASD suggests a possible, unanticipated, inverse relationship.

**Conclusions:**

Exploring the relationship of disturbed sleep to behavior and symptom severity in children with ASD can provide an understanding of how poor sleep influences ASD symptoms. This study identified distinct differences in ASD symptom severity between and within individual participants and found unique, and unexpected, symptom patterns. This finding supports the need, in research and treatment, to identify comorbidities and symptoms that contribute to individual behavioral profiles and phenotypes of the disorder.

## Background

Regardless of developmental level, sleep problems in children contribute to emotional and behavioral delays. (1) Pediatric sleep problems may include sleep disordered breathing, sleep apnea, insomnia, sleepwalking, and parasomnias. (2) Disturbed sleep can impair cognitive and executive functioning by decreasing a child’s mental flexibility, inhibition, and working memory. (3) Even if sleep problems resolve, negative effects on cognition and behavior may continue years after the sleep problem has resolved. (4, 5) Sleep problems in children that persist may have detrimental effects on neurological development and adversely impact the child’s well-being. (6, 7)

Sleep problems are more prevalent in children with autism spectrum disorder (ASD). (8, 9) Sleep disorders have been reported in 56–80% of children with ASD. (10, 11) Parents of children with ASD report that their children have more difficulty falling asleep, sleep less, and wake up more often than reported by parents of neurotypical peers as well as reports of typically developing siblings. (12–14) Parents of children with ASD also report their children have more sleep-associated anxieties and parasomnias than typically developing children. (9, 13, 15)

Sleep studies in children have shown more erratic sleep patterns in children with ASD than with typically developing peers. (6, 16) Children with ASD exhibit decreased sleep efficiency (decreased time asleep/time in bed), decreased sleep duration and continuity, and more frequent night awakenings accompanied by long periods of wakefulness not experienced by neurotypically developing children. (5, 17–19)

Children with ASD and poor sleep are more likely to experience increased levels of sensory sensitivity, hyperactivity, and anxiety than those with ASD and no sleep disturbances. (10, 17, 20) Similarly, parents who report increased sleep disturbances in their children with ASD also note that their children exhibit more ritualistic and compulsive behavior than do parents of children with ASD and no sleep disturbances. (12, 21) Children with increased bedtime rituals or compulsive behavior may show increased bedtime resistance or be unable to adapt to changes in their environment or routine.

Previous studies specifically examining the relationship between sleep and behavior in ASD are limited. A long-term study (20,000 nights, n = 67) of children and adolescents with low functioning ASD in a residential facility confirmed that prolonged disturbed sleep increased agitation, tantrums, and destructive behavior for this cohort. (22) Sleep disturbances have been associated with poor adaptive functioning and more severe ASD symptoms. (14, 20) This research supports the role of disturbed sleep for initiating and/or exacerbating these and other ASD associated behaviors. Research into the diverse clinical presentations of ASD may contribute to better understanding as well as effective, targeted treatments of ASD symptoms.

The purpose of this descriptive, correlational study was to examine and describe the patterns of sleep in children with autism spectrum disorder (ASD) and explore the relationship between disturbed sleep and problem behaviors common to ASD. To this researcher’s knowledge, this is first study to examine sleep patterns and ASD-specific behavioral patterns in an outpatient setting.

## Methods

### Design

This study employed a descriptive correlational, observational design to examine children’s sleep characteristics and their relationship to ASD symptoms using actigraphy and a parent-completed sleep diary.

### Setting and Sample

Participants were recruited from a large urban and suburban geographical area included in a tertiary hospital system database. Flyers were posted on social media and at in-person events held by local autism support groups. Eligible participants were children between the ages of 6–12 with a medically conferred diagnosis of ASD, as reported by a parent or found in the hospital database. Actigraphy measurement took place in the child’s home and not during an extended school break. Parents and children had to be willing to participate in the study activities of actigraphy collection at night. Parents agreed to complete the sleep diaries each night and morning for the duration of study and refrain from taking long trips or collecting data while the child was sleeping in unfamiliar setting. Parents consented to avoiding changing medications and therapy regimes for the duration of data collection.

Children were excluded from study if they were medically unable to participate, had a change in medication or therapy in the past 21 days, or had a reported or documented history of seizures in the past six months or a history of regressive onset ASD (lost early language and social skills previously obtained). Eligible participants were consented and evaluated in the research facility or in their home.

### Data Collection

Following Institutional Review Board (IRB) approval for the study, eligible participants who responded to the flyers or emails were contacted by phone and invited to come to the university or offered an in-home visit to learn about the study and provide written informed consent. Parents were asked to provide a basic history of the child’s medical conditions, medical symptoms, and current medications. They completed the *Autism Spectrum Rating Scale* (ASRS) parent response forms (23) and were given instructions on completing the sleep diary and having the child wear the GT3X actigraph. (24)

Actigraphy measurements and corresponding sleep diary data were collected for up to seven nights. Data collection occurred during regular school semesters, as recommended in previous actigraphy studies. (13, 25) Of the seven-night duration, a minimum of four nights of measurable data was required to be included in the analysis. If the parent reported that the child was ill, the usual sleep pattern was disturbed, or the child did not sleep at their home, the night was removed from analysis. If the investigator determined by the report that the actigraph was not worn or removed during the night, the data were removed. Children unable to tolerate wrist actigraphy for any time period were given shirts with a pocket on the shoulder for actigraph placement.

Sleep disturbances, reported by the parent on the sleep diary, were collected for their child for up to seven days. Instructions for wearing the actigraph included the option to wear the actigraph continuously throughout the day and night or to place the actigraph on the child’s wrist (or shirt pocket) 10–30 minutes prior to bedtime until thirty minutes after waking.

### Measures

A sleep disturbance scale was created for this study using a combination of actigraphy and parent-completed sleep diary reports. The number of sleep disturbances per night (increased latency, wake multiple times, extended wakefulness, early morning wake) were collected and quantified providing a value from 0–4 for each participant. A score of 0 indicates no nighttime sleep disturbance and higher values indicate one or multiple sleep disturbances.

The parent report sleep diary (26) included an indication of the time the child was put down for bed, the observed time sleep was initiated, the number of times the child woke up during the night, the number of minutes the child was awake during the night, the number of hours the child slept, and the time the child got out of bed in the morning. Additionally, caregivers could report any additional sleep disturbances they observed and list situational or environmental factors that may have disrupted sleep or data collection.

### Actigraphy

The actigraph (produced by Actigraph_tm_, Pensacola, FL) was placed on the child 30 minutes prior to attempting to initiate sleep. The actigraph captured the time the child fell asleep, the amount of time asleep, the number of arousals during the night, the time and duration of wake through the night, and time of wake in the morning. The total sleep time divided by the time allotted for sleep (captured by the sleep diary) was used as the measure of sleep efficiency (SE).

Sleep was defined as 10 consecutive minutes of immobility. Sleep onset was defined as the time of the first minute of 10 consecutive minutes of immobility captured by the actigraph. Morning wake was the time of the last minute of sleep occurring in the last 10 consecutive minutes of immobility captured by the actigraph.

### Autism Spectrum Rating Scale

The *Autism Spectrum Rating Scale* (ASRS) was used to quantify parents’ observations of children’s behavioral symptoms associated with ASD. The ASRS uses a five-point Likert rating scale for parents and teachers/caregivers to evaluate how often they observe specific behaviors in the child or adolescent, with scores equal to and greater than 60 indicative of impairment. This evaluation includes assessments of impairments in the use of language, unusual or repetitive behaviors, tolerance of changes associated with routine, emotional responses and reactions to sensory stimulation, attention and impulse control, and motor control. (23) For analysis, ASRS questions are grouped into three categorical symptom scores: social/ communication, unusual behavior, and self-regulation. Scores on the ASRS are based on DSM-5 diagnostic criteria, and the results include a score in each of the forementioned categories as well as an overall DSM-5 severity score. Reverse coded items were corrected prior to analysis.

### Data Analysis

Descriptive analysis was used to examine the sleep diary, actigraphy, and ASRS results. Average time and standard deviation of sleep onset latency, wake after sleep onset frequency and duration, early morning wake time, total nighttime sleep, and sleep efficiency were determined. Data from actigraphy were used as the primary source of information for the study. Data missing from the actigraphy report were substituted with data from the sleep diary (e.g., if wake time not captured due to an equipment failure, the time noted on the sleep diary was used). If data were missing and could not be resolved, the night was removed from the analysis.

Data were collected by actigraphy for up to seven nights. Data from actigraphy were used as the primary measure of sleep pattern variables. Missing data from actigraphy were substituted with available sleep diary data for one participant that could not tolerate the device on their wrist nor on their clothing.

Parents could report if the child was sick or had an unusual night (such as a sleepover or forgetting to wear the actigraph). If the information from that night was significantly different from the rest of the collection period, it was discarded. The diary was also used to resolve discrepancies in actigraph data. If a discrepancy was unable to be resolved, that night’s data were discarded.

Of the participants that could tolerate the device, data were collected for 4–7 nights with an average of 6.43 nights (SD = .82) per participant. The study was powered based on a two-tailed test. It was determined that to explore the relationship between the number of sleep disturbances and severity of ASD symptoms, a sample size of 24 with an alpha of .05 and a power of .80 would produce a medium-to-large effect size of .54.

## Results

### Demographic Characteristics of the Study Sample

Forty-one families of children with ASD responded to the study flier and/or email recruitment. Of these 41 families, 29 were eligible for study. Since the study was designed to represent the sleep patterns of participants during the school year, enrollment was discontinued due to the state’s mandatory closing of schools at the start of the COVID-19 pandemic. Prior to school closings, 24 participants had completed the study and were included in this analysis.

Demographic characteristics of the participants are presented in Table 1. Of the 24 participants with ASD studied, 75% (n = 18) were male. The age of study participants ranged from 6 to 12 years of age, and the mean age of participants was 9.2 years (SD = 1.72). Parents were asked to provide a brief medical history for their child (Table 1). Four children participating in this study had previously been diagnosed with a sleep disorder upon enrollment in the study. The most common secondary neurological diagnoses reported by parents were attention deficit and hyperactivity disorder (ADHD), anxiety, and delayed speech. Less common diagnoses included non-verbal communication, obsessive compulsive disorder (OCD), intellectual disability, and depression. All 24 parents reported that their child experienced altered responses to sensory information (over or under responsivity to touch, taste, smell, sound, or light).

### Actigraph Tolerance

Overall, 96% of the children studied provided usable actigraphy data. Of the 24 participants, 67% (n = 16) tolerated the wrist actigraphy for the duration of the study. Seven participants (29%) wore the actigraph on their shirt for sleep, and only one participant (4%) could not tolerate the device on their wrist or their nighttime clothing. Fourteen children (58%) provided actigraphy data for seven full nights, six (25%) provided six nights of usable data, two (8%) provided five nights of useable actigraphy data, and one child (4%) wore the device for four nights of data collection.

### Sleep Pattern Demographics

Twenty-two children experienced at least one and as many as four symptoms of disturbed sleep. Table 2 describes the means and standard deviations of sleep characteristics for the entire study population, including those with no sleep disturbances.

Minutes of disturbed sleep reported in the sleep diary by the parents of the child who did not wear the device were used to include this participant in the study correlative analyses. Estimated minutes of sleep latency, wake after sleep onset (WASO), and early morning wake, as well as the estimated WASO number provided by the family, are not included in the actigraphy measures for this participant in Table 2. This participant was also excluded from the sleep efficiency and total night sleep (TNS) calculation.

Mean sleep efficiency of study participants was 85.2% with a wide range of 80–96% and a standard deviation of 4.84. Mean total sleep time was 492.67 minutes, or 8 hours and 12 minutes. Night sleep time ranged from 5 hours and 37 minutes to 9 hours and 22 minutes (SD = 53.34 minutes).

Table 3 demonstrates the means and standard deviations of specific disturbed sleep symptoms identified in the 22 participants who experienced sleep disturbances. Mean sleep latency, identified as a sleep disturbance exceeding 30 minutes (n=7) was greater than 65 minutes with a range of 30–180 minutes. Of the 17 children experiencing WASO, mean nighttime awakenings was 21.07 with a range of 15–35. Those staying awake for longer than five minutes had a mean wake time of 7.1 minutes and a range of 5–15 minutes per wake episode. Participants who woke before 5:30 a.m. were awake for an average of 15.64 minutes before 5:30 a.m. (range for this early wake was 5–37.5 minutes). Seven participants experienced early morning awakening.

Although the actigraph was the primary source of data for this study, the sleep diary was utilized if actigraphy was unavailable. Fortunately, this secondary source was utilized for only one participant. Table 4 displays parent-reported sleep diary data for all 24 participants. Although parent report is considered reliable, discrepancies were observed in parent-reported numbers of WASO as opposed to what was captured by the actigraph. In the sleep diaries, parent-recorded wake times were significantly shorter than the time captured by the actigraph.

### Behavioral Demographics

The study sample contained only participants with a diagnosis of ASD and as expected, all participants demonstrated increased scores on the ASRS (see Table 5) indicating ASD and social and behavioral impairments consistent with ASD. ASRS data were available for all 24 participants.

Of the 24 participants, 23 (96%) scored over 60 on the ASRS DSM scale, indicating symptom severity for the ASRS questionnaire is consistent with a diagnosis of ASD. Only one participant (4%) scored in an average range (indicating typical levels of concern) with a score of 59, the highest value in that category. This individual had an average score for unusual behavior and elevated scores for both the severity of social communication delays and self-regulation.

Two participants (8%) scored in the average range for social communication, three participants (13%) scored in the average range for unusual behavior, and six participants (25%) had average scores for self-regulation. Two of the three participants with average scores in unusual behaviors also had average scores in self-regulation and scores over 60 in DSM severity. This supports the presence of communication delays severe enough to qualify the child for a diagnosis of ASD, although unusual behaviors and the ability to self-regulate were not rated as problematic by the parent (at least at the time of this assessment).

The mean score for symptoms matching DSM criteria was 70.33 (SD = 5.85) with a range of scores from 59 to 84. This range indicates inclusion of both low- and high-functioning individuals with ASD in the study sample.

Table 6 displays the mean, range, and standard deviations of the ASRS results. Elevated scores indicate more severe impairment in that category. Impairments in social and communication skills were most frequently reported in this population. Scores indicating severity of self-regulation impairments were the lowest symptom scores in this study population.

Cronbach’s alpha for the subscales of the DSM score for social/communication, unusual behavior, and self-regulation items were acceptable, .84-.86 in verbal participants (n=21) and .83-.85 in non-verbal participants (n=3). This is slightly lower than in previous studies (.86-.95) (Table 7).

### Inter-relationships

Pearson r analysis supported a significant positive correlation between the number of sleep disturbances and the severity of social and communication delays (r = .59, p <.01). Although it did not reach statistical significance (p = .07), the effect size (r = −.38) between the number of sleep disturbances and the unusual behavior score suggests the presence of an inverse relationship between these two variables (see Table 8). No statistically significant relationship was found between the number of sleep disturbances and self-regulation impairment scores (r = −.29, p = .17).

In addition to determining that the number of sleep disturbances affected social and communication delays, an analysis of the relationship between each specific type of sleep pattern disturbance with the social/communication T-score was explored to assess for a relationship between specific sleep disturbance symptoms and social and communication delays. Pearson r calculation determined that an average wake time of greater than five minutes through the night was significantly correlated with social and communication delays (r = .59, p < .01). The correlation of early morning wake and social and communication delays was also significant (r = .51, p = .01). Although tested, the sleep disturbances of delayed sleep latency and waking more than 15 times through the night did not have a significant relationship to delays in social and communication skills (Table 9).

## Discussion

Children with ASD present with variable combinations of social, behavioral, and communication impairment. Reasons behind these varied presentations remain poorly understood and are important areas of research. In this study, participants had a more severe presentation of social and communication delays or unusual behaviors, but a higher score in one category did not determine a higher score in another (Table 10).

Although sleep disturbances are common in this population and impact the daily function of children and adults without ASD, this study did not support a relationship between the number of sleep disturbances and the severity of diagnostic ASD symptoms. However, an increased number of sleep disturbances was positively related to delays in social and communication skills. Given the results of previous studies (Johnson et al., 2018; Mazurek & Sohl, 2016; Sauders et al., 2009), a positive relationship was expected between the number of sleep disturbances and the presence of unusual behaviors and delays in self-regulation in children with ASD but was not supported by data from this sample. This study did not find a significant correlative relationship with either ASD symptom; in fact, these data suggest the possible presence of a negative trend.

This bidirectional finding supports consideration of a relationship between sleep disturbances and social and communication delays but sleep disturbances did not appear to contribute to unusual behaviors in ASD. Severe scores for both delays were present in this sample and, independently, had a correlative relationship with a more severe diagnostic score of ASD (Table 10). Evaluating comorbidities and symptom clusters specific to children with severe behavioral delays, separately from those experiencing severe social delays, may aid in the treatment of individual persons with ASD and further contribute to the description of ASD phenotypes.

### Strengths and Limitations of the Study

This study included families from a midwestern U.S. city and its eastern, western, and southern suburbs and was limited to subjects with ASD; comparative data were not collected on children that did not have a diagnosis of ASD. Participants were recruited locally from support networks and the children’s hospital. Recruitment was most successful from word-of-mouth of active participants and from computer-generated emails sent to parents of patients meeting study criteria within the hospital system. The hospital system had over 1,900 email addresses of families of potential participants with ASD who met the age requirement and had not had a recent medical history of seizure disorders. A random list of 500 emails was provided for study recruitment. Emails were sent in batches of 10–30 per week during the enrollment period. Due to the closing of schools across the state resulting from the pandemic, recruitment was suspended after only 160 emails were sent.

The number of children in this study sample with sleep disturbances was higher than reported in previous studies of children with ASD. It is possible that families with ASD children who have sleep disturbances were more interested in participating in a sleep study. The study was fortunate to have at least two participants in each of the five categories of sleep disturbances and a varying range of severity in each of the symptom categories. To reduce variance, the sample was limited to a single age range and controlled for a specific ASD onset.

Variability of the data for participants in this study contributed to significant results for one research question. The number of sleep disturbances had a positive correlation with social communication severity and was adequately powered. The medium-to-large effect size of a possible negative correlation between the number of sleep disturbances and the severity of unusual behavior scores presents an intriguing future area of study.

A post-hoc analysis of this finding generated a power of .49 in a two-tailed test (.62 in a one-tailed test with an alpha of .05). Unfortunately, with a small sample size, it is difficult to determine how individual age and medical differences contributed to study findings. Future research into this relationship, performed with larger samples, may support a polarized symptom profile in a child with ASD.

## Conclusions

In summary, sleep of 24 children with various degrees of ASD severity were assessed, and results demonstrated that multiple sleep disturbances were present. Most participants, with ASD, experienced disturbed sleep and the number of sleep disturbances positively correlated with the severity of social and communication delays in ASD. A significant finding was expected between the number of sleep disturbances and the severity of behaviors in ASD but was not present. These results, in fact, suggested an inverse relationship and further contributes to the theory of distinct ASD phenotypes and the need for targeted research and treatment.

## Figures and Tables

**Table 1:** Demographic Characteristics of the Sample (N=24).

Characteristics	N (%)	Mean	SD	Range

Gender				

Male	18 (75%)			

Female	6 (25%)			

				

Age (years)	N = 24	9.21	1.719	6–12

6	2 (8.3%)			
7	3 (12.5%)			
8	3 (12.5%)			
9	3 (12.5%)			
10	7 (29.2%)			
11	5 (20.8%)			
12	1 (4.2%)			

Medical History				
Autism Spectrum Disorder	24 (100%)			

Intellectual Disability	2 (8.3%)			

Non-verbal	3 (12.5%)			

Delayed Speech	16(66.7%)			

Attention Deficit/ Hyperactivity Disorder (ADHD)	17(70.8%)			

Seizure Disorder	1 (4.2%)			

Sleep Disorder	4 (16.7%)			

Depression	1 (4.2%)			

Anxiety	12 (50%)			

Obsessive Compulsive Disorder	3 (12.5%)			

Motor Disturbances	2 (8.3%)			

Non-food Allergies	6 (25%)			

Other	5 (20.8%)			

Altered Sensory Processing	24 (100%)			

GI Disturbances				
Food Sensitivity	7 (29.2%)			
Constipation	11(45.8%)			
Diarrhea	1 (4.2%)			
Reflux	2 (8.3%)			
GI Other	2 (8.3%)			

**Table 2:** Characteristics of Sleep Patterns in Children with ASD (N=24).

Characteristics	N (%)	Mean	SD	Range

Number of Sleep Disturbances	24	1.67	1.13	0–4
0	2 (8.3%)			
1	12(50.0%)			
2	4 (16.7%)			
3	4 (16.7%)			
4	2 (8.3%)			

Sleep Latency (minutes)	23	26.4	38.98	0.2–180

WASO (average number of times awake per night)	23	17.7	8.32	4–35

WASO (minutes awake after sleep onset per night)	23	69.49	30.3	15–117

Early Morning Wake (minutes awake before 5:30 a.m.)	23	4.5	9.4	0–37.5

Sleep Efficiency	23	85.2	4.84	80–96%

Total Time Asleep at Night (TNS) (minutes) (hh:mm)	23	492.67 (08:12)	53.34	338–562 (05:37–09:22)

Nights Collected (number of nights of usable actigraph data per participant)	23	6.43	.82	4–7

*Note.* WASO: wake after sleep onset

**Table 3:** Characteristics of Disturbed Sleep Symptoms in Children with ASD (N=22).

Characteristics	N (%)	Mean	SD	Range	SE % (Range)
Sleep Latency (>30 mins)	7 (29.2)	65.19 min	54.13	30–180	84 (80–94)
WASO (more than 15 times)	17 (70.8)	21.07 times	6.89	15–35	83 (80–89)
Extended Wake (>5 min average wake time)	9 (37.5)	7.1 min	3.11	5–15	83 (80–86)*
Early Morning Wake (minutes before 5:30 a.m.)	7 (29.2)	15.64 min	11.74	5–37.5	85 (80–91)

*Note.* WASO: wake after sleep onset; SE: sleep efficiency

* Calculation based on n = 8 participants who wore the actigraphy watch and experienced this sleep disturbance.

**Table 4:** Results of Information Collected from Sleep Diaries.

Sleep Diary Variable	N	Mean	Range
Number of Times WASO	24	1.7	0–8
Number of Minutes WASO	24	35.2	0–240

*Note.* WASO: wake after sleep onset

**Table 5:** Number of Participants in Each Category of DSM Autism Severity and Symptom Severity (N=24).

		N (%)			
T-Score	Classification	DSM	SC	UB	SR
					
70+	Very Elevated (Many more concerns than are typically reported)	11 (46)	8 (33)	12 (50)	6 (25)
65–69	Elevated Score (More concerns than are typically reported)	11 (46)	9 (38)	6 (25)	4 (17)
60–64	Slightly Elevated Score (Somewhat more concerns than are typically reported)	1 (4)	5 (21)	3 (13)	8 (33)
40–59	Average Score (Typical levels of concern)	1 (4)	2 (8)	3 (13)	6 (25)
< 40	Low Score (Fewer concerns than are typically reported)	0	0	0	0
					

*Note.* DSM: Diagnostic Manual of Mental Disorders, SC: social and communication score, UB: unusual behavior score, SR: Self-regulation score

**Table 6:** Results of Autism Spectrum Rating Scale (ASRS) (N=24).

Impairment	N	Mean	Range	Standard Deviation
DSM Severity *T*-score	24	70.33	59–84	5.85
Social Communication *T*-score	24	67.79	57–84	7.28
Unusual Behavior *T*-score	24	68.21	56–77	5.59
Self-Regulation *T*-score	24	63.88	50–76	6.39

*Note.* DSM: Diagnostic Manual of Mental Disorders

**Table 7:** Cronbach’s Alpha for Outcome Variables (N=24).

Variable	Number of Items	Reliability Cronbach’s alpha	
Current Study	Previous Literature		
Verbal Participants (n = 21)			
DSM	40	.84	.91–.95
Social Communication	19	.86	.91–.95
Unusual Behavior	24	.85	.87–.95
Self-Regulation	17	.84	.86–.92
Non-verbal Participants (n = 3)			
DSM	34	.83	.91–.95
Social Communication	17	.85	.91–.95
Unusual Behavior	18	.83	.87–.95
Self-Regulation	16	.83	.86–.92

*Note.* DSM: Diagnostic Manual of Mental Disorders

**Table 8:** Correlation Matrix of Number of Sleep Disturbances (0–4) and DSM ASD Symptom Severity in Children with ASD (N=24).

Variable	Number of Sleep Disturbances (N=24)	
	Correlation coefficient (r)	*p*-value
Social Communication *T*-score	**.59** **	**<.01**
Unusual Behavior *T*-score	−.38	.07
Self-Regulation *T*-score	−.29	.17

** Correlation is significant at the .01 level (2-tailed)

**Table 9:** Correlation Matrix of Specific Sleep Disturbances and Social and Communication Delays in Children with ASD (N=24).

Variable	Social Communication *T*-score	
	Correlation coefficient (r)	*p*-value
Extended Wake (>5 minutes)	**.59** **	**<.01** **
Sleep Latency	.30	.15
Wake After Sleep Onset (>15 times)	−.01	.98
Early Morning Wake (before 05:30)	**.51** *	**.01** *

* Correlation is significant at a level < 0.05 (2-tailed).

** Correlation is significant at a level < 0.01 (2-tailed).

**Table 10:** Correlations Among the ASRS Scales (N=24).

Variable	DSM	SC	UB	SR
DSM	–			
SC	**.49** *	--		
UB	**.69** **	−.19	--	
SR	.15	−.27	.38	--

*Note.* DSM: Diagnostic Manual of Mental Disorders, SC: social and communication score, UB: unusual behavior score, SR: Self-regulation score

* Correlation is significant at the 0.05 level (2-tailed).

** Correlation is significant at the 0.01 level (2-tailed).

**Table 11:** ASRS Scores and Number of Sleep Disturbances Grouped by Age (N=24).

Age (in years)	N	DSM Range (mean)	SC Range (mean)	UB Range (mean)	SR Range (mean)	NSD Range (mean)
6	2	68–69 (68.5)	63–68 (65.5)	68–70 (69)	60–66 (63)	1–1 (1)
7	3	61–75 (70.3)	66–84 (72.3)	57–74 (63.3)	50–70 (57.6)	0–3 (2.5)
8	3	71–76 (73.3)	57–73 (65)	71–74 (72)	68–71 (70)	0–2 (1.6)
9	3	68–73 (70)	68–79 (72.6)	64–71 (68.3)	58–68 (61.3)	1–2 (1.3)
10	7	65–76 (69.1)	57–72 (64.1)	61–73 (69)	55–76 (63.4)	0–3 (1.4)
11	5	59–82 (68.4)	60–81 (68.8)	56–74 (65.6)	62–70 (64.8)	1–4 (2.6)
12	1	NA* (84)	NA* (73)	NA* (77)	NA* (72)	NA* (1)

*Note.* DSM: Diagnostic Manual of Mental Disorders, SC: social and communication score, UB: unusual behavior score, SR: Self-regulation score, NSD: number of sleep disturbances

* There is no range for children aged 12 years; only one child was in this category.

## Data Availability

The data that support the findings of this study are available from University Hospitals Cleveland Medical Center (UHCMC), but restrictions apply to the availability of these data, which were used under license for the current study, and so are not publicly available. Data are however available from the authors upon reasonable request and with permission of UHCMC.

## Abbreviations

ASD: autism spectrum disorder
IRB: Institutional Review Board
ASRS: Autism Spectrum Rating Scale
DSM/DSM-5: Diagnostic and Statistical Manual of Mental Disorders, fifth edition
ADHD: attention deficit and hyperactivity disorder
OCD: obsessive compulsive disorder
WASO: wake after sleep onset
TNS: total night sleep
SE: sleep efficiency
SC: social and communication
UB: unusual behavior
SR: Self-regulation
NSD: number of sleep disturbances
